# Plasma Exchange for Refractory Pruritus Due to Drug-Induced Chronic Cholestasis Following Azithromycin Misuse in COVID-19 Infection

**DOI:** 10.7759/cureus.60884

**Published:** 2024-05-22

**Authors:** Newnex Mongare, Kelvin Orare, Swafiya Busaidy, Ahmed Sokwala, Christopher Opio

**Affiliations:** 1 Internal Medicine, Aga Khan University, Nairobi, KEN; 2 Medicine, Aga Khan University, Nairobi, KEN

**Keywords:** covid-19, azithromycin, cholestasis, refractory pruritus, plasma exchange

## Abstract

Azithromycin can result in severe cholestatic liver disease. We describe two cases of intractable pruritus secondary to drug-induced cholestatic liver injury, unresponsive to symptomatic medical therapy, necessitating and responding well to therapeutic plasma exchange (TPE). The first is a case of a 60-year-old male known to have stable chronic lymphocytic leukemia (CLL) and benign prostatic hyperplasia, and the second is a 46-year-old female known to have primary biliary cirrhosis (PBC) who presented at six weeks and two weeks, respectively, post-mild-COVID-19 pneumonia. Their drug histories were positive for over-the-counter (OCT) azithromycin use during the COVID-19 pneumonia period. They presented with a two-week history of severe itching, associated with sleep deprivation and impaired quality of life. Liver function tests revealed a cholestatic pattern of liver injury. Pruritus remained refractory to multiple lines of treatment including bile acid sequestrants and antihistamines. Rapid and long-lasting relief of the patient’s symptoms was observed after three sessions of TPE. Our cases highlight medically recalcitrant cholestatic pruritus as an adverse effect of antibiotic misuse during the recent COVID-19 pandemic. Sustained symptomatic improvements were seen after TPE.

## Introduction

COVID-19 is an infectious viral illness caused by the severe acute respiratory syndrome - coronavirus 2 (SARS-CoV-2), whose presentation ranges from an asymptomatic process to a mild flu-like illness, to a severe inflammatory condition that leads to multi-organ failure and death [1]. Due to the global impact of the COVID-19 pandemic, there was a thought to repurpose known drugs to combat the disease. Azithromycin was considered one of the possible therapeutic options given its immune-modulating effect, antiviral effect, and antibacterial effect in community-acquired pneumonia [2]. However, high-quality evidence has disproved the role of azithromycin in COVID-19 [3].

Despite this, over-the-counter (OTC) misuse of azithromycin to treat mild COVID-19 pneumonia remains prevalent [4]. This not only increases the risk of antimicrobial resistance but increases the risk of developing debilitating adverse reactions [5]. Azithromycin’s side effect profile ranges from mild symptoms such as nausea, abdominal pain, diarrhea, dyspepsia, and headache to severe adverse events that include Clostridia difficile diarrhea, QTc prolongation, and hypersensitivity reactions such as Stevens-Johnson syndrome and toxic epidermal necrolysis. Few case reports describe cholestatic hepatotoxicity as an adverse effect of azithromycin [6]. SARS-CoV-2, although more frequently occurring in severe COVID-19 cases, has also been shown to be hepatotoxic [7]. Despite a dearth of mechanistic studies exploring SARS-CoV-2’s effects on hepatocytes and the consequences of using potentially hepatotoxic drugs, such as azithromycin, in the setting of COVID-19 pneumonia, drug and disease may interact to worsen liver injury outcomes [8].

Herein, we describe two cases of severe cholestatic pruritus after azithromycin use for mild COVID-19 infections, unresponsive to symptomatic medical therapy, necessitating and responding well to therapeutic plasma exchange (TPE).

## Case presentation

Case A

**Figure 1:**
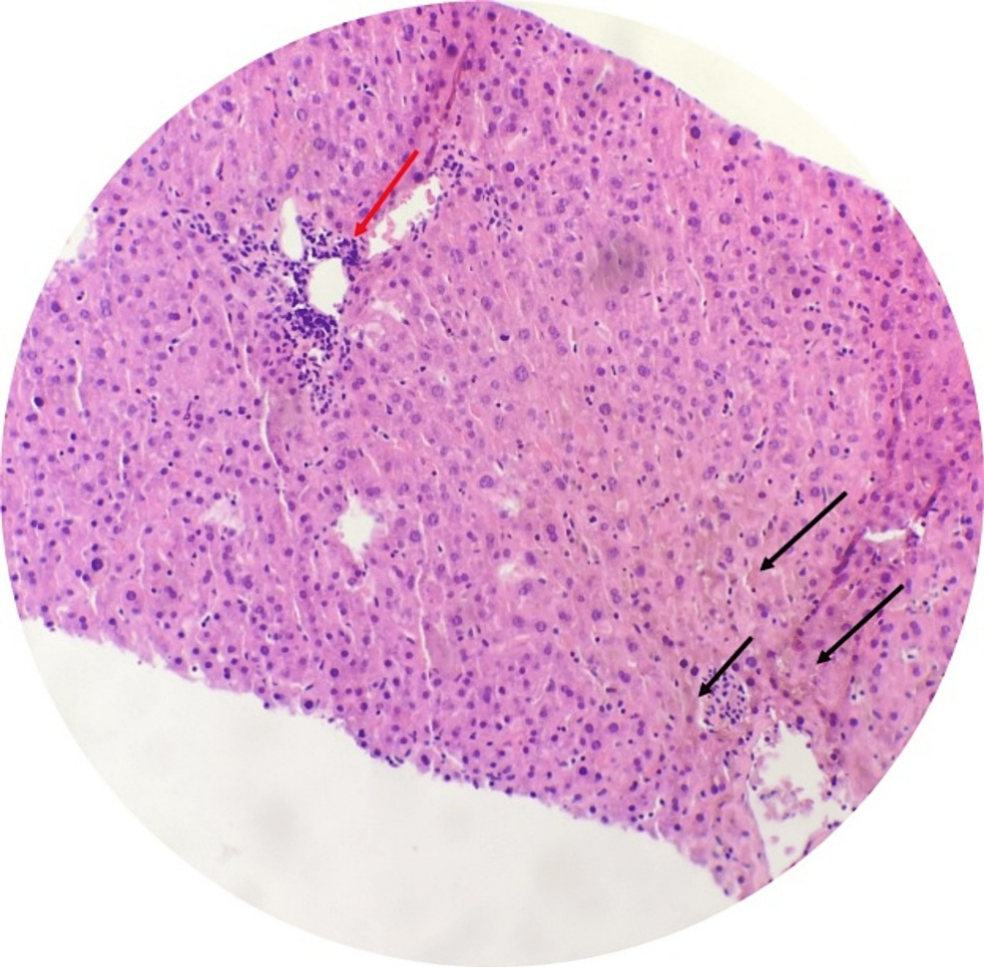
Liver core biopsy (hematoxylin and eosin) Parenchyma shows normal architecture; there are focal collections of lymphocytes within the periportal area (red arrow) and cholestasis (black arrows) (×40).

A 60-year-old male patient presented with a history of progressively worsening yellowness of eyes, severe generalized pruritus, pale stool, and darkened urine. The pruritus was associated with sleep deprivation and a significant decline in his quality of life. His past medical history was significant for chronic lymphocytic leukemia (CLL) on watchful waiting and benign prostatic hyperplasia doing well on medical management (Tamsulosin). Six weeks before his admission, he had been diagnosed with mild COVID-19, for which he self-isolated at home and completed five days of OTC azithromycin 500 mg daily. On examination, he had normal vitals. He was deeply jaundiced with mild right upper quadrant tenderness. His laboratory investigations demonstrated an elevated alkaline phosphatase of 220 U/L (46.00-116.0 U/L), gamma-glutamyl transferase of 27 U/L (0.00-73.00 U/L), total bilirubin of 407 µmol/L (5.0-21.0 µmol/L), direct bilirubin of 323 µmol/L (0.0-5.0 µmol/L), aspartate transaminase of 59 U/L (0.00-34.00 U/L), and an alanine transferase of 126 U/L (0.00-49.00 U/L). His calculated ratio (R) factor of liver injury was 1.7, consistent with a cholestatic pattern of injury. An extensive infectious and autoimmune liver panel was unremarkable. A computerized tomography scan abdomen showed extensive retroperitoneal, mesenteric, and porta hepatis lymphadenopathy with mild splenomegaly. A hepatobiliary iminodiacetic acid scan showed a normal liver. However, there was minimal tracer uptake precluding evaluation of the biliary tracts. He underwent a magnetic resonance cholangiopancreatography (MRCP), which revealed no obstructing lesion, no intrahepatic biliary dilation, no strictures, and no filling defects in the biliary tree. We went on to perform a liver biopsy that revealed hepatic parenchyma with mild chronic inflammation and lobular cholestasis, which suggested cholestatic hepatitis as shown in Figure 1.

The patient had a protracted inpatient stay, with multiple lines of medical therapy being tried unsuccessfully. This included antihistamines (hydroxyzine 12.5 mg mane and 25 mg nocte), ursodeoxycholic acid (450 mg thrice daily), and a trial of steroids (methylprednisolone 40 mg once daily) without any improvement. The patient still had significantly disturbed sleep, with the patient now frustrated by the futility of care. Throughout his admission, he had consistently rated his itch intensity as a 10 on a numerical rating scale (NRS) that ranged from 0 (“no itch”) to 10 (“worst imaginable itch”) [9]. A decision was made to attempt TPE. The exchange was scheduled to run over four hours with a blood flow of 200 mL/min to be replaced with 3 L of fluid (constituted as 200 mL of albumin and 800 mL of ringer’s lactate). Heparin was used. By the following day, the patient was already reporting significant improvement in his symptoms, with his itch score dropping to 7. He went on to have three additional alternate-day TPE sessions, with the itch score reaching a nadir of five out of 10 after the second session and stagnating there. One week after his last TPE session, the patient’s score remained five, with the patient, however, endorsing significant improvements in life quality and sleep.

Case B

A 48-year-old female presented with a two-week history of severe pruritus that was worse at night. Her itch score was 10/10. Her past medical history was significant for being followed up for primary biliary cirrhosis (PBC) for the last five years, which was well controlled. She had also been diagnosed with mild COVID-19 pneumonia, two weeks before admission, for which she admitted to using OTC azithromycin 500 mg daily for five days. On examination, her vitals were within normal limits with an unremarkable systemic examination except for pruritic marks all over her skin. Her laboratory evaluation demonstrated deranged liver function tests; total bilirubin of 45 µmol/L (5.0-21.0 µmol/L), direct bilirubin of 41 µmol/L (0.0-5.0 µmol/L), alkaline phosphatase of 179 U/L (46.00-116.0 U/L), gamma-glutamyl transferase of 275 U/L (0.00-73.00 U/L), aspartate transaminase of 87 U/L (0.00-34.00 U/L), and alanine transaminase of 115 U/L (0.00-49.00 U/L). She also had a cholestatic pattern of liver injury with a ratio (R) factor of 1.9. Despite being on multiple lines of medical treatment for pruritus including ursodeoxycholic acid 300 mg twice daily, bile acid sequestrants (cholestyramine 4 g twice daily), fibrates (bezafibrate 400 mg daily), and an antihistamine (promethazine 12.5 mg nocte), there was no improvement in her itch score. She was initiated on TPE on day two of admission. Her itch score improved after two sessions of TPE reaching a nadir of five out of 10. She was discharged after three TPE sessions, and her itch score remained at five out of 10 during her two-month follow-up.

## Discussion

Antibiotic misuse during the SARS-CoV-2 pandemic has been a major concern, with azithromycin being one of the antibiotics misused OTC at the community level despite high-quality evidence disproving its role [4,10]. This can unnecessarily expose the patient to debilitating and sometimes fatal adverse drug effects. Azithromycin-induced idiosyncratic hepatotoxicity, albeit rarely, has been reported. All three phenotypes of drug-induced liver injury (DILI), that is, hepatocellular, cholestatic, and mixed phenotypes have been described with azithromycin, with hepatocellular injury being the most predominant pattern of injury [6,11]. The mechanisms that underlie azithromycin-induced liver damage have not been elucidated, but it is thought to be a metabolite effect [12].

In both our cases, the patients notably had stable co-morbidities that may have predisposed them to cholestatic liver injury, that is, CLL and PBC. It remains unclear whether preexisting liver injury can increase the risk of idiosyncratic drug reactions; however, they have been known to develop more severe liver injury [13]. In addition, both patients were recovering from mild COVID-19 pneumonia. Post-COVID-19 cholangiopathy is a novel entity that is being reported in the literature. Although most case reports are documenting the phenomenon in patients with critical or severe COVID-19, COVID-19 infection, however mild, may have increased the susceptibility of the patients to azithromycin-induced DILI [14].

Similar to previously reported cases, the onset of the symptoms was within two to three weeks of azithromycin use (6). Most patients, however, exhibit benign clinical features with a self-limiting course that includes jaundice, abdominal pain, nausea, pruritus, rash, and fever. Rarely, do patients exhibit fatal or chronic liver injury. Our cases remain noteworthy in that intractable itching and refractory to standard medical therapy were the predominant presentations with attendant significant morbidity. Additionally, unlike other case reports, merely stopping the medication was not enough to cause the resolution of symptoms.

Pruritus is a known complication in people with cholestatic liver disease from any cause such as PBC, primary sclerosing cholangitis, and even drug-induced cholestasis. It is an irritating symptom that can significantly impact one’s quality of life [15]. The mechanism of pruritus in cholestasis is diverse and complex; however, it is largely understood to be secondary to increased circulating levels of pruritogens such as bile acids, lysophosphatidic acid (LPA), endogenous opiates, and progesterone derivatives [16].

TPE has been described in the literature to be a useful measure in the management of refractory pruritus. It is believed to work by removing the pruritogens from the plasma [17]. It is safe and effective, with a prospective cohort study done among PBC with refractory cholestatic pruritus reporting a decrease in their NRS itch score from 8.3 to 3.1 (p<0.0001), with the antipruritic effects persisting throughout a 90-day follow-up period [18]. We demonstrate that TPE can similarly be employed to ameliorate refractory drug-induced cholestatic pruritus.

## Conclusions

Pruritus is a disabling and frequent symptom of liver disease and can affect patients' quality of life. Azithromycin is a rare but potential cause of debilitating cholestatic pruritus. Treatment of pruritus frequently involves different modalities. Our cases demonstrate the challenges in the management of refractory pruritus and highlight the role of TPE.
